# Hydrogen evolution reaction from bare and surface-functionalized few-layered MoS_2_ nanosheets in acidic and alkaline electrolytes

**DOI:** 10.1016/j.mtchem.2019.100207

**Published:** 2019-12

**Authors:** B. Lai, Subhash C. Singh, J.K. Bindra, C.S. Saraj, A. Shukla, T.P. Yadav, W. Wu, S.A. McGill, N.S. Dalal, Amit Srivastava, Chunlei Guo

**Affiliations:** a The Institute of Optics, University of Rochester, Rochester, NY, 14627, USA; b Department of Chemistry and Biochemistry, Florida State University, Tallahassee, FL, 32306, USA; c Changchun Institute of Optics, Fine Mechanics and Physics, Chinese Academy of Sciences, Changchun, 130033, China; d Department of Physics, Institute of Science, Banaras Hindu University, Varanasi, 222005, India; e National High Magnetic Field Laboratory, Florida State University, Tallahassee, FL, 30201, USA; f Department of Physics, TDPG College, VBS Purvanchal University, Jaunpur, 222001, India

**Keywords:** Hydrogen evolution reaction, Oxygen evolution reaction, Acidic and alkaline electrolytes, Molybdenum disulfide, Multiwalled carbon nanotubes, Ferromagnetic electrocatalysts

## Abstract

Hydrogen is considered as an ideal and sustainable energy carrier because of its high energy density and carbon-free combustion. Electrochemical water splitting is the only solution for uninterrupted, scalable, and sustainable production of hydrogen without carbon emission. However, a large-scale hydrogen production through electrochemical water splitting depends on the availability of earth-abundant electrocatalysts and a suitable electrolyte medium. In this article, we demonstrate that hydrogen evolution reaction (HER) performance of electrocatalytic materials can be controlled by their surface functionalization and selection of a suitable electrolyte solution. Here, we report syntheses of few-layered MoS_2_ nanosheets, NiO nanoparticles (NPs), and multiwalled carbon nanotubes (MWCNTs) using scalable production methods from earth-abundant materials. Magnetic measurements of as-produced electrocatalyst materials demonstrate that MoS_2_ nanoflakes are diamagnetic, whereas surface-functionalized MoS_2_ and its composite with carbon nanotubes have strong ferromagnetism. The HER performance of the few-layered pristine MoS_2_ nanoflakes, MoS_2_/NiO NPs, and MoS_2_/NiO NPs/MWCNT nanocomposite electrocatalysts are studied in acidic and alkaline media. For bare MoS_2_, the values of overpotential (η_10_) in alkaline and acidic media are 0.45 and 0.54 V, respectively. Similarly, the values of current density at 0.5 V overpotential are 27 and 6.2 mA/cm^2^ in alkaline and acidic media, respectively. The surface functionalization acts adversely in the both alkaline and acidic media. MoS_2_ nanosheets functionalized with NiO NPs also demonstrated excellent performance for oxygen evolution reaction with anodic current of ~60 mA/cm^2^ and Tafel slope of 78 mVdec^−1^ in alkaline medium.

## Highlights

• Facile and scalable synthesis of few-layered MoS_2_ nanosheets and its surface-functionalized derivatives.
• Switching from diamagnetism to strong ferromagnetism through surface functionalization.
• Hydrogen evolution reaction in alkaline and acidic electrolyte.
• Higher HER performance of few-layered MoS_2_ nanosheets and its surface-functionalized derivatives in alkaline medium.

## Graphical abstract

**Figure:**
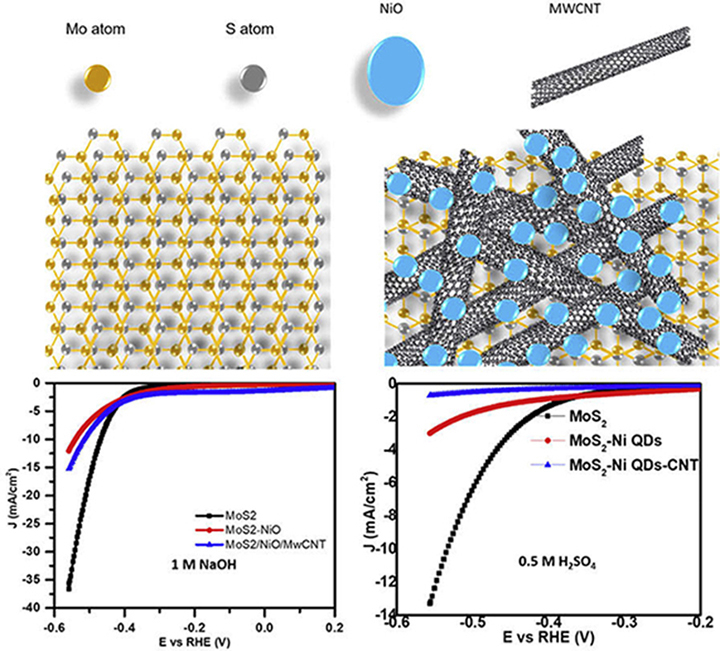

## 1 Introduction

Global energy demand is rising continuously and predicted to increase by 57% by the year 2050 [1]. The development of green, renewable, and affordable energy solutions that can fulfill global energy requirements with zero carbon emission is an acute and significant issue of our society. Hydroelectric, geothermal, solar, wind, and other renewable energy sources are considered promising carbon-neutral alternatives to fossil fuels. However, hydrothermal and geothermal sources have geographical constraints that require long-distance transmission, whereas solar, wind, and other renewable energy sources are often intermittent and require large-scale storage to be practical for grid integration [2]. Transmission and storage challenges associated with conventional renewable energy sources demand an alternative clean energy solution. Hydrogen is considered as an ideal and sustainable energy carrier because of its high energy density, clean combustion, and applications in the production of commodity chemicals, such as ammonia, and refining of petroleum and metals [3,4]. Currently, fossil fuel reformation is the primary hydrogen production method. However, this process consumes a large amount of energy with significant carbon emission [5,6]. One of the most efficient and green alternatives to producing H_2_ is reductive half reaction of electrochemical water splitting known as hydrogen evolution reaction (HER: 2H^+^ + 2e = H_2_), where hydrogen is produced at the cathode in the presence of an appropriate electrocatalyst [7,8]. Platinum is well recognized and acknowledged as the best-performing electrocatalyst because of its high chemical and corrosion resistance and low overpotential to drive the reaction. However, its scarcity and high cost limit its wide application in the industrial-scale H_2_ production. Thus, the search for earth-abundant, non-precious metals for high-efficiency HER with long-term stability in an electrolyte solution has attracted significant attention [[9], [10], [11]]. A range of earth-abundant electrocatalysts including non-noble metal [12], transition metal dichalcogenides [[13], [14], [15]], carbides and nitrides [16], phosphides [17,18], sulfides [19], layered hydroxides [20], selenides [21], and their composites have been used for HER [[12], [13], [14], [15], [16], [17], [18]] and as electrode materials [21].

Recently, molybdenum disulfide (MoS_2_), a quasi 2D transition metal dichalcogenide, has been considered as an efficient non-noble metal electrocatalyst for HER because of the presence of active sulfur atoms [22,23]. MoS_2_ nanomaterials and their composites have attracted significant attention from researchers because of their novel electronic, optical, optoelectronic, and catalytic properties [24,25]. The bulk MoS_2_ is an indirect bandgap semiconductor with band gap energy of 1.29 eV where multiple layers of S–Mo–S are stacked together with weak van der Waals interactions [26]. Surface functionalization of MoS_2_ with electron-withdrawing or electron-donating atoms, ions, molecules, clusters, and nanomaterials can control its catalytic/photocatalytic properties and stability in an electrolyte solution [27,28]. For example, the functionalization of MoS_2_ with electron-donating molecules increase its stability in an electrolyte solution and allows it to operate at lower overpotential [29]. Molecules or clusters adsorbed on the surface or used to functionalize the surface can provide additional sites for the adsorption of hydrogen and/or these can weaken hydrogen-binding among water molecules for easier splitting [30]. Furthermore, molecules or clusters can modify the electronic properties of MoS_2_ that facilitates charge transfer between host and guest to increase HER activity [27].

Electrolytes are an important component in electrochemical reactions where the reaction rate and selectivity of different electrocatalytic processes, such as charge transfer at electrode–electrolyte interface and stability of electrocatalyst materials, depend on species present in the electrolyte and its pH [31,32]. Electrocatalysts get dissolved into acidic media because of leaching of metal ions into the electrolytes, while electrochemical reactions make a layer of metal hydroxide when operating in an alkaline medium [31]. Comparative study of HER performance of pristine and surface-functionalized MoS_2_ in acidic and alkaline media is necessary to choose the better electrocatalyst and electrolyte system for optimized, long-lasting, and a low-cost production of hydrogen.

Here, we report syntheses of few-layered MoS_2_ nanosheets, NiO nanoparticles (NPs), and multiwalled carbon nanotubes (MWCNTs) using scalable production methods from earth-abundant materials. Magnetic measurements of as-produced electrocatalyst materials demonstrate that MoS_2_ nanoflakes are diamagnetic, whereas surface-functionalized MoS_2_ and its composite with carbon nanotubes have strong ferromagnetism. The HER performance of the few-layered pristine MoS_2_ nanoflakes, MoS_2_/NiO NPs, and MoS_2_/NiO NPs/MWCNT nanocomposite electrocatalysts are studied in acidic and alkaline media. For bare MoS_2_, the values of overpotential (η_10_) in alkaline and acidic media are 0.45 and 0.54 V, respectively. Similarly, the values of current density at 0.5 V overpotential are 27 and 6.2 mA/cm^2^ in alkaline and acidic media, respectively. The surface functionalization acts adversely in the both alkaline and acidic media. The MoS_2_ nanosheets functionalized with NiO NPs also demonstrated excellent performance for oxygen evolution reaction (OER) with anodic current of ~60 mA/cm^2^ and Tafel slope of 78 mVdec^−1^ in alkaline medium. Present research shows that the alkaline medium is more suitable for MoS_2_ and its surface-functionalized derivatives for electrocatalytic hydrogen generation.

## 2 Materials and methods

### 2.1 Materials

N-dimethylformamide (DMF), nickel acetate [Ni (CH_3_CO_2_)_2_·2H_2_O], sodium hydroxide (NaOH), and sulfuric acid (H_2_SO_4_) were procured from Sigma-Aldrich. All the reactants and solvents were of analytical grade and used as received without further purification. The aqueous solutions were prepared by using deionized water and ultrapure double-distilled water, as and when required.

### 2.2 Synthesis of electrocatalytic materials

**Fig. 1:**
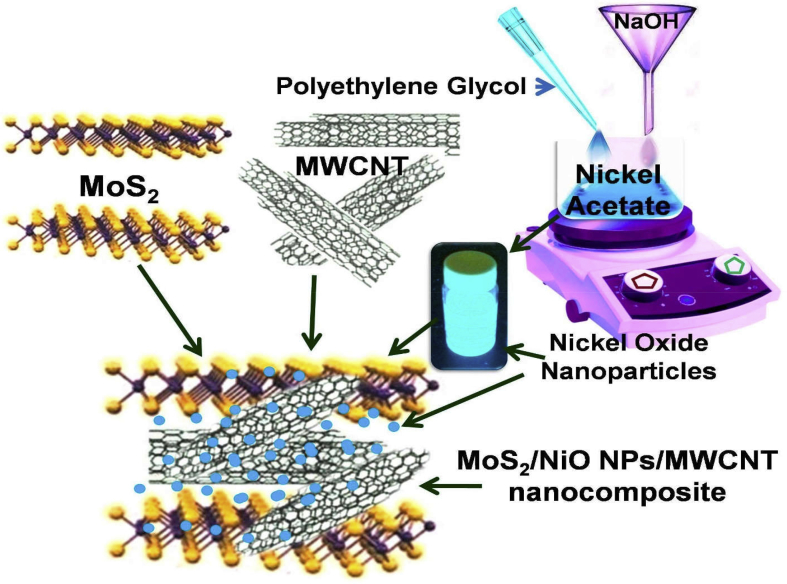
Schematic illustration for the synthesis of MoS_2_/NiO NPs/MWCNT nanocomposite.

The synthesis process of the incumbent materials involved the following steps. Synthesis protocol is schematically presented in Fig. 1.

#### 2.2.1 Synthesis of multiwalled carbon nanotubes

The MWCNTs were synthesized through chemical vapor deposition (CVD) technique on the Al–Cu–Fe surface, and ethylene (C_2_H_4_) was used as a carbon source in the process. Briefly, the CVD chamber was first evacuated and heated under the ambience of argon and hydrogen (Ar/H_2_10:1) mixture at the pressure of ~250 mbar. The MWCNTs were grown at 1072 K in approximately 20 min. Subsequently, the furnace was switched off and allowed to cool down to near room temperature under the argon atmosphere. The black deposition inside the quartz tube was taken out and thoroughly washed first with HCl:HNO_3_ (1:3 ratio) for 10 min followed by with distilled water for 60 min.

#### 2.2.2 Synthesis of nickel oxide nanoparticles

Nickel oxide NPs were prepared through a chemical reduction of nickel acetate with polyethylene glycol as a stabilizing agent. In a typical synthesis procedure, 1 M aqueous solution of nickel acetate was first mixed with polyethylene glycol under continuous stirring for 60 min. An aqueous solution of 1 M NaOH was filled into a burette tube in the vertical column and dispensed drop by drop into nickel acetate/PEG mixture under continuous stirring. The resultant solution was centrifuged at 5000 rpm for 10 min with the addition of 200 ml of deionized water and finally stored in the glass vial for further use.

#### 2.2.3 Synthesis of few-layered molybdenum disulfide

Few-layered MoS_2_ nanosheets sample was synthesized by a mechanical exfoliation of bulk MoS_2_ powder (purity 99.999% Sigma Aldrich) in dimethyl formamide (DMF) solvent using a high-intensity exfoliation in a pressurized ultrasonic reactor. Briefly, 50 mg MoS_2_ powder was suspended in 500 ml DMF and exfoliated under intensive ultrasonication for 10 h. Two milliliters supernatant of NiO NPs as obtained from 2.2.2 was added in the MoS_2_/DMF solution and further exfoliated for next 10 h. Two milligrams of as-prepared and cleaned MWCNTs powder obtained in the step 2.2.1 was added in the MoS_2_/DMF/NiO NPs and homogenized under continuous magnetic stirring for 50 h. Finally, the obtained solutions were filtered using 0.22 μm porous filter membranes and subsequently washed with deionized water for several times. The final sample was dried in vacuum at 80 °C for 12 h and stored in cleaned airtight glass vials for further characterization and applications.

### 2.3 Characterization of electrocatalysts

Copper oxide (CuO; 99.95%), selenium oxide (SeO2; 99.97%), silver oxide (AgO; 99.97%), sodium hydroxide (NaOH; 99.97%), polyvinylpolymide (PVP; MW 8000), and ethylene glycol (EG) were purchased from Alfa Aesar and were used without further puriﬁcation.

#### 2.3.1 Synthesis procedure deionized water and a mixture of deionized water, PVP and EG mixture were used as solvents for the synthesis of diﬀerent samples

In a typical synthesis procedure for samples S1–S3, 1.6 g CuO and 1.11 g SeO2 powders were added into 36 ml water followed by ultrasonic dispersion for 30 min to make the ﬁrst solution. In a separate glass vessel, 0.8 g of NaOH was dissolved into 100 ml of deionized water to make a 0.2 M solution. Both solutions were transferred into 150 ml eﬂon-lined stainless-steel autoclave and maintained at a constant re-action temperature (150–260 °C) for 24 h followed by natural cooling. Products were separated by centrifugation, washed 2–3 times sequen-tially with water and ethyl alcohol, dried at 60 °C in an air oven and ﬁnally stored in dried and cleaned glass vials for further characteriza-tions and applications. For samples S4–S7, mixture of 0.5 g poly-vinylpyrrolidone (PVP) into 36 ml of EG was used as a solvent in plac Materials Copper oxide (CuO; 99.95%), selenium oxide (SeO2; 99.97%), silver oxide (AgO; 99.97%), sodium hydroxide (NaOH; 99.97%), poly-vinylpolymide (PVP; MW 8000), and ethylene glycol (EG) were pur-chased from Alfa Aesar and were used without further puriﬁcation.

#### 2.3.2 Optical, structural, vibrational, compositional, and surface morphological characterizations

The samples were characterized by using different analytical techniques. As obtained electrocatalyst powders were ultrasonically dispersed in double-distilled water to make a colloidal solution. UV–visible absorption spectra of water-dispersed electrocatalysts samples were recorded using PerkinElmer Lambda 365 double-beam spectrophotometer. PANalytical X'Pert PRO X-ray diffractometer with CuK_α_ radiation source (λ = 1.54178 Å) was used to diagnose crystallinity and phase of different catalysts powder using X-ray diffraction in the 2θ range of 10̊-80̊. The surface composition and chemical environment of the as-synthesized samples were studied using PHI 5000 X-ray photoelectron spectroscopy with AlK_α_ X-ray source. The energy calibrations were made against the C1s peak. A silicon chip was used to hold the sample. Renishaw invia microRaman spectrometer with 584 nm excitation line from Ar–Kr laser was used for Raman spectroscopic measurements. A 100× objective lens was used to focus laser beam (~1 μm spot size) on the sample surface and collection of back scattered Raman signal. The data were recorded with a 10 s accumulation time. To get an insight of the surface morphology and microstructures of as-synthesized electrocatalyst samples, scanning electron microscopy (FEI Nova 400, operated at 20 kV) and transmission electron microscopy (JOEL 2100F, operated at accelerating voltage of 200 kV) studies were carried out.

#### 2.3.3 Magnetic property measurements

Magnetization measurements for electrocatalyst samples were performed on a Quantum Design superconducting quantum interference device (SQUID) magnetometer. Zero-field-cooled (ZFC) and field-cooled (FC) measurements were carried out under a constant magnetic field of 100 Oe in the temperature range of 1.8–300 K. Isothermal field-dependent magnetization measurements were conducted with magnetic field varying in the range of 0–7 T at 1.8 K. Sample holders were measured separately under identical conditions, and their magnetic responses were subtracted directly from the raw data.

### 2.4 Electrochemical measurements

Electrocatalyst ink of each catalyst sample were prepared by first ultrasonic dispersion of 5 mg of each powder sample into 1 ml of DMF solution followed by the addition of 50 μl of Nafion solution (0.5 M) into mixture as adhesive and further ultrasonication for 1 h. The working electrode was fabricated by deposition of 4 μl of thus prepared catalyst ink on a glassy carbon electrode (3 mm diameter) followed by drying under a tungsten lamp. Electrochemical measurements were performed on BioLogic VMP3 multichannel workstation with a three-electrode system, where a Pt wire, a catalyst-loaded glassy carbon electrode, and a saturated calomel electrode (SCE) were used as counter, working, and reference electrodes, respectively. Working electrode, connected with motor, was allowed to spin with 500 rpm. Aqueous solutions of 0.5 M H_2_SO_4_ and 1 M NaOH were used as acidic and alkaline electrolytes for electrochemical measurements. Linear sweep voltammetry (LSV) curves were measured by sweeping voltage in the range of 0.2 to −0.8 V (*versus* SCE) for acidic medium and −0.6 to −1.6 V (V *versus* SCE) for alkaline medium with the scan rate of 10 mVs^−1^. Expression $E_{RHE}=\;E_{SCE}+E_{SCE}^{0}+0.0592*pH$, where $E_{SCE}^{0}=0.242V,$was used to translate V *versus* SCE to V versus reverse hydrogen electrode (RHE). Working electrodes were first prestabilized in the electrolyte solution using 30–60 scans of cyclic voltammetry before performing LSV measurements. For both electrolytes, Electrochemical Impedance Spectra (EIS) were recorded with the biasing of working electrode at – 0.5 V (vs RHE) and superimposing a small alternating voltage of 10 mV over the frequency range of 10 mHz–1 MHz. The potential axes of polarization curves were iR-corrected, where R is corresponding impedance value at 10^4^ Hz. The cyclic voltametry (CV) measurements were recorded in non-Faradaic region from −0.2 to +0.2 V (SCE) with different scan rates 10 mV/s, 20 mV/s, 40 mV/s, 60 mV/s, 80 mV/s and 100 mV/s in order to get double-layer capacitance (C_dl_) and total active surface area (ESCA). The LSV curves for working electrodes made of MoS_2_ were measured after 1000 CV cycles in acidic and alkaline media to test their stabilities.

## 3 Results and discussion

### 3.1 UV–visible absorption, X-ray diffraction and Raman spectroscopic measurements

**Fig. 2:**
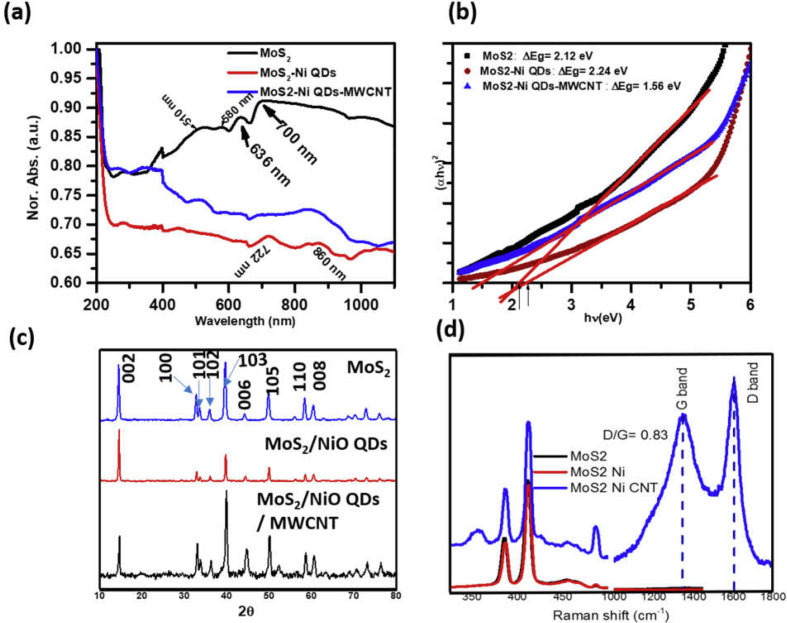
(a) UV–visible absorption, (b) corresponding Tauc's plots, (c) X-ray diffraction, and (d) Raman spectra of MoS_2_, MoS_2_/NiO NPs and MoS_2_/NiO NPs/MWCNT nanocomposite.

UV–visible absorption spectra of water-dispersed electrocatalysts are presented in Fig. 2(a)**.** Absorption peaks at 510, 580, 636, and 700 nm are characteristics of few-layered MoS_2_ with trigonal prismatic configuration. A sharp peak at 636 nm and a hump at ~700 nm are characteristic peaks associated with the direct excitonic transition at the K point of the Brillouin zone [33,34]. Broader absorption peak around 510 nm is due to the electronic transition from deeper valance electronic band to the conduction band. Functionalization of few-layered MoS_2_ with NiO NPs reduces Mie scattering at longer wavelength and characteristic absorption peaks became clearer with a slight red shift indicating an interaction between NiO quantum dots (QDs) and MoS_2_ flakes (red curve). With the addition of MWCNT into NiO QDs–functionalized MoS_2_, two well-separated absorption peaks at ~722 nm and ~860 nm convoluted together to make a broader peak centered at ~850 nm. The absorption coefficient, α, of water-dispersed power samples under Beer's law, is related to their corresponding bandgap energy following the expression $\alpha =A{\left(h\nu -E_{g}\right)}^{n}/h\nu$, where A is a constant, E_g_ defines band gap of the material, and the exponent n may have values ½, 2, 3/2, and 3 for allowed direct, allowed indirect, forbidden direct, and forbidden indirect transitions, respectively [35,36]. Few-layered MoS_2_ is known to be a direct bandgap semiconductor [33,34]; therefore, we used n = 1/2 in the present study to calculate band gap of different electrocatalyst samples. The bandgap energies of different samples are obtained by the intercept of linear fitting of ${\left(\alpha h\nu \right)}^{2}\;$*versus* hν graph, well known as Tauc's plot, at the hν axis (Fig. 2(b)). The calculated bandgap energy for pristine few-layered MoS_2_ is 2.12 eV (Fig. 2 (b)), which is larger than the direct band gap (1.8 eV) reported in the literatures for few-layered MoS_2_ nanosheets [31]. Larger bandgap energy may be due to the quantum confinement of few-layered MoS_2_ nanosheets from lateral directions. Surface functionalization of few-layered MoS_2_ nanosheets with NiO NPs further increases bandgap energy from 2.12 to 2.24 eV either because of (i) the formation of smaller sized flakes in the presence of NiO NPs or (ii) NiO NPs perturb electronic energy level associated with the top of the valance band and/or bottom of the conduction band. However, the mixing of MWCNT into MoS_2_/NiO QDs decreases the bandgap energy from 2.24 to 1.56 eV. Decrease in the bandgap energy may be associated with the formation of one or more energy levels in the band gap or shift in the band edge due to the interaction of carbon atoms at the surface of MWCNT with sulfur atoms in MoS_2_ flakes.

Fig. 2(c) displays XRD patterns of the as-synthesized pristine MoS_2_ flakes, Ni QD–functionalized MoS_2_ flakes, and MoS_2_/NiO QDs/MWCNT nanocomposite. Each sample exhibits a strong diffraction peak at 2θ = 14.1° corresponding to Bragg reflection from (002) plane, with interlayer (d) spacing of 0.614 nm, indicating a typical lamellar structure along the c axis. In addition, several weaker reflections from the (100), (101), (102), (103), (006), (105), (110), and (008) planes are observed at higher diffraction angles, demonstrating the polycrystalline characteristic of 2H–MoS_2_ (JCPDS 37–1492). The intensity of diffraction peak from a plane is a consequence of constructive interference of diffracted X-ray signal from different aligned crystal planes. Therefore, a decrease in the intensity of diffraction peak corresponding to (002) reflection plane of MoS_2_/NiO sample demonstrates fewer layers over the pristine MoS_2_ [35]. Well-separated absorption peaks at 722 nm and 860 nm (Fig. 2(a), red curve) also supports this claim. Further decrease in the intensity of (002) diffraction peak in the MoS_2_/NiO/MWCNT nanocomposite demonstrates that the surface of MWCNTs are functionalized with fewer layers of NiO-decorated MoS_2_ nanoflakes. In other words, we can say that fewer layers of MoS_2_ nanoflakes exhibit a larger number of active sites to bind with the surface of MWCNTs. Absence of additional diffraction peaks in MoS_2_/NiO and MoS_2_/NiO/MWCNT electrocatalysts samples demonstrates that MoS_2_ nanoflakes are still a major constituent in all the electrocatalyst and NiO NPs and MWCNTs control electronic and catalytic properties of MoS_2_ nanoflakes.

Raman spectroscopy has been used to further confirm the structural features of MoS_2_, MoS_2_/NiO NPs, and MoS_2_/NiO NPs/MWCNT nanocomposites (Fig. 2(d)). The appearance of peaks at 287.7, 384.4, and 410.3 cm^−1^ corresponds to E_1g_, E^1^_2g_, and A_1g_ vibrational modes of hexagonal pure 2H–MoS_2_ (Fig. S1). As we know, the E^1^_2g_ and A_1g_ vibrational modes are associated with the atomic displacements those are orthogonal to each other. Here, A_1g_ corresponds to out-of-plane symmetric displacement of sulfur atoms along the c-axis, whereas the E^1^_2g_ involves in-plane displacement of Mo and S atoms. The E_1g_ and E^1^_2g_ are linked with the relative vibrational mode along the layer of the bond between Mo and S atoms. Surface functionalization of few-layered MoS_2_ with NiO NPs causes a shift in vibrational modes toward larger wavenumber side (Fig. 2 (d)**;** Fig. S1). It means that smaller-sized NiO NPs may get intercalated between MoS_2_ layers during synthesis that reduces interatomic interaction between Mo and S atoms from two adjacent layers. The Raman spectrum of the MoS_2_/NiO NPs/MWCNT nanocomposite exhibits lower wavenumber shift with respect to MoS_2_/NiO NPs sample, but larger wavenumber shift over the pristine MoS_2_ nanosheets. This may be either due to the reduced intercalating effect of NiO NPs with the addition of MWCNTs or formation of covalent bond between active sulfur atoms at the surface of MoS_2_ with carbon atoms at the surface of MWCNTs. Later one has better possibility because of strong affinity of making covalent bond between carbon atoms at the surface of MWCNT and sulfur atoms from MoS_2_ edge. Furthermore, the Raman spectrum of MWCNT feature the D band at 1349.5 cm^−1^ corresponding to the phonon scattering from local defects or disorders present in the CNTs, and the G band at 1604.0 cm^−1^ associated with in-plane tangential stretching of the C–C bonds in the graphitic structure (Fig. 2(d)**:** blue curve).

### 3.2 Scanning electron microscopy (SEM) and transmission electron microscopy (TEM) of different electrocatalyst samples

**Fig. 3:**
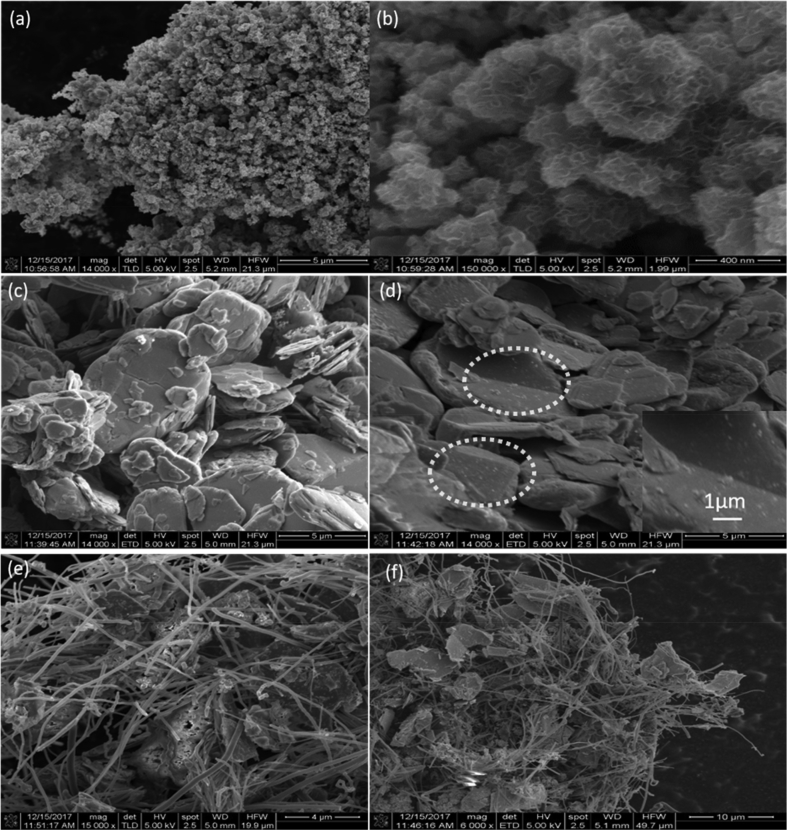
SEM images of (a,b) MoS_2_, (c,d) MoS_2_/NiO NPs and (e,f) MoS_2_/NiO NPs/MWCNT nanocomposite**.**

**Fig. 4:**
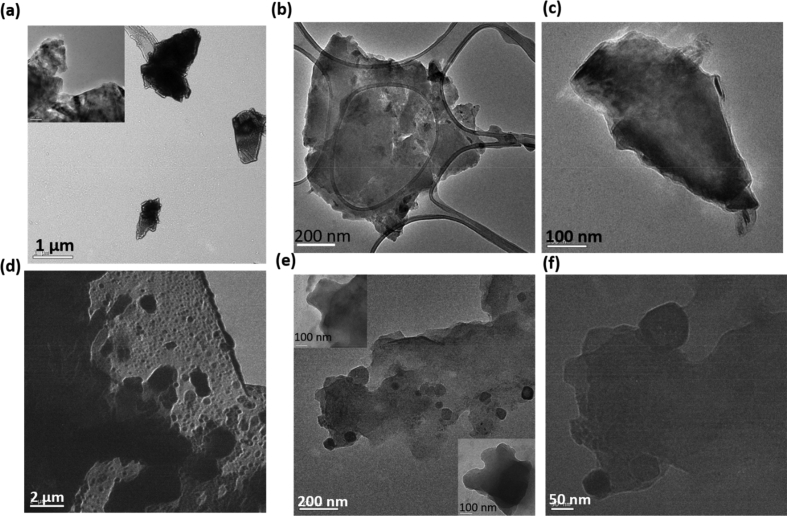
TEM images of (a–c) MoS_2_ nanoflakes at scale bar (a) 1 μm (b) 200 nm, and (c) 100 nm, and (d–f) MoS_2_/NiO NPs at the scale bar of (d) 2 μm, (e) 200 nm (top left and bottom right insets: an enlarged view of a nanoflake at the scale of 100 nm and (f) 50 nm.

SEM and TEM images of pristine and surface-functionalized MoS_2_ nanosheets are presented in Fig. 3 and Fig. 4 respectively. Pristine MoS_2_ nanoflakes get assembled into 3D space to form free standing flower like architectures (Fig. 3(a) and (b)**).** The thickness of each of the free-standing flakes lies in the range of 4–10 nm. Ultrasonic dispersion of MoS_2_ nanoflakes into double-distilled water disassembles flower-like architecture into constituent nanoflakes of size 1–2 μm (TEM images: Fig. 4(a–c)). Addition of NiO NPs into ultrasonic reactor significantly altered the morphology of as-produced MoS_2_ flakes. Here, 2D nanoflakes of 1–5 μm lateral dimensions staked together to form thick sheets. A large number of smaller-sized (0.5–1 μ) MoS_2_ flakes are attached to the surface of larger-sized flakes (white encircled region). TEM images of NiO NP–functionalized MoS_2_ nanosheets are presented in Fig. 4(d–f). Similar to pristine MoS_2_, ultrasonic dispersion of NiO/MoS_2_ stacks (Fig. 3(c) and (d)) also disintegrates them into their constituent smaller flakes (Fig. 4(d–f)). It is clear from TEM images (Fig. 4(e) and (f)) that NiO NP_S_ of 20–50 nm are functionalized on the surface of MoS_2_ nanoflakes. SEM images of MoS_2_/NiO NPs/MWCNT nanocomposite are shown in Fig. 3(e and f). Here, smaller 2D flakes of 1–4 μm are attached with MWCNT of 250–500 nm diameter.

### 3.3 X-ray photoelectron spectroscopic investigation of different electrocatalysts

**Fig. 5:**
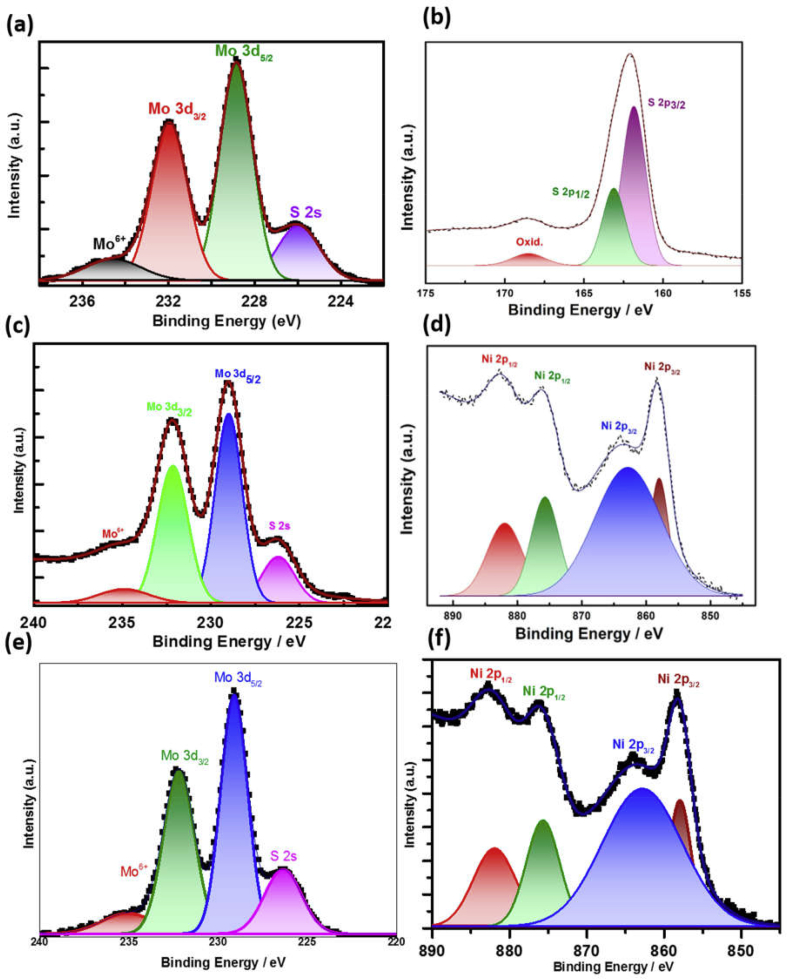
XPS spectra of (a,b) MoS_2_, (c,d) MoS_2_/NiO NPs, and (e,f) MoS_2_/NiO/MWCNT nanocomposite.

High-resolution X-ray photoelectron spectroscopy (XPS) measurements have been performed to gain further insights into the chemical character and bonding states of the MoS_2_, MoS_2_/NiO, and MoS_2_/NiO/MWCNT nanocomposites. The peaks depicted in Fig. 5(a–f) and Fig. S2 confirm the presence of main elements in as-synthesized MoS_2_, MoS_2_/NiO, and MoS_2_/NiO/MWCNT nanocomposites, particularly signals related to Mo, S, Ni, C, and O. In the pristine MoS_2_ sample (Fig. 5(a)**),** a doublet peak of Mo 3d appears at 228.5 and 231.6 eV, which are attributed to Mo 3d_5/2_ and Mo 3d_3/2_ oxidation states, respectively. It primarily owes to Mo^4+^ oxidation state in the pristine MoS_2_ and has been in accordance with the XPS data in the previous report [38]. The appearance of an additional weak peak at ~234.17 eV corresponding to the Mo^6+^ oxidation state shows presence of MoO_3_ as trace [39]. The photoelectron peak appeared at 225.9 eV ascribes to the sulfur 2s state. Moreover, deconvoluted peaks of S 2p at 162.3 and 163.4 eV correspond to the atomic states S 2p_3/2_ and S 2p_1/2_ for S 2p (Fig. 5(b)**),** which is due to presence of sulfur element in the sulfide.

Furthermore, to give insightful direct evidence, the chemical composition of NiO NP–functionalized MoS_2_ has been analyzed by XPS. As observed in Fig. 5(c–d), the MoS_2_/NiO mainly comprises Mo, S, and Ni elements. Fig. 5(c) exhibits two characteristic peaks of Mo 3d_3/2_ and Mo 3d_5/2_ at 232.2 and 229.0 eV, respectively, which is indicative of the dominance of Mo^4+.^ The contribution from the peak centered at 234.9 eV ascribes to Mo^6+.^ The Ni 2p doublets at around 862.1, 868.2, 879.9, and 886.1 eV confirm the presence of Ni^2+^ in NiO NPs (Fig. 5(d–f)) displays the high-resolution core-level XPS spectra of the as-synthesized MoS_2_–NiO/MWCNT nanocomposites Gaussian-fitted for Mo, S, Ni, C, and O. Fig. 5(e) illustrates the XPS spectrum of MoS_2_–NiO/MWCNT nanocomposite sample in 220–240 eV. A doublet present at 229.2 and 232.2 eV corresponds to Mo^4+^-3d_5/2_ and Mo^4+^-3d_3/2_ components of MoS_2_, respectively. A careful observation of the deconvolution of these peaks reveals the presence of some additional strong peak which have been shifted to higher binding energies by ~0.6 eV with respect to the position of the Mo3d peaks from pristine MoS_2_. Furthermore, the relatively weak peak detected at 235.2 eV corresponds to the Mo^6+^ oxidation state. Moreover, the Ni 2p doublets at around 858.2 and 864.0 eV of Ni-2p_3/2_ and 876.1 and 882.1 eV of Ni-2p_1/2_ confirm the presence of Ni^2+^ as shown in Fig. 5(f). These findings indicate that the as-synthesized MoS_2_/NiO NPs/MWCNT composite possesses a very high concentration of metallic MoS_2_. Fig. S2 shows the carbon C1s XPS spectrum from MoS_2_/NiO/MWCNT nanocomposite. Gaussian deconvolution of C1s XPS peak demonstrates that it is a convolution of six different energy peaks. The main peak centered at 284.6 eV represents a standard C peak [40], whereas the peak at 286.2 eV indicates the presence of the C atoms bound to the oxygen atoms, which is originated because of the nitric acid treatment of MWCNT surface with a small number of oxygen-containing functional groups.

### 3.4 Magnetic characterizations of different electrocatalysts

**Fig. 6:**
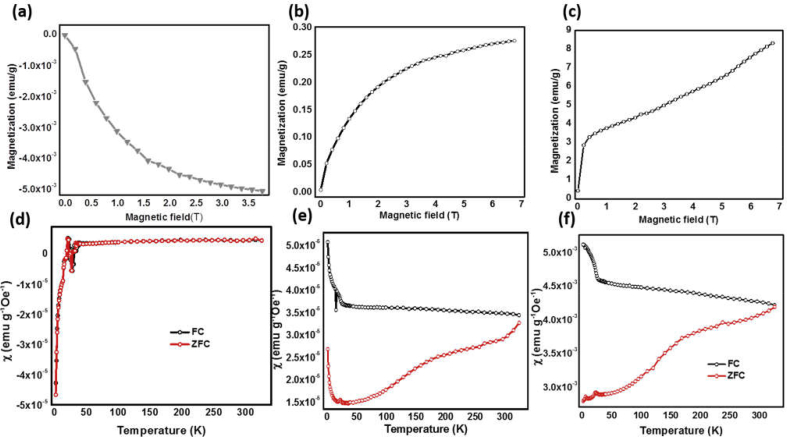
Magnetization (M) *versus* magnetic field (T) curves of (a) pristine MoS_2_, (b) NiO NP–functionalized MoS_2_, and (c) MoS_2_/NiO NPs/MWCNT nanocomposite. Field-cooled (FC) and zero-field-cooled (ZFC) curves for (d) pristine MoS_2_, (e) NiO NP–functionalized MoS_2_, and (f) MoS_2_/NiO NPs/MWCNT nanocomposite.

Magnetic functionality in electrocatalyst materials may provide additional control for electrochemical reaction at the electrode–electrolyte interface using an external magnetic field. Catalyst material dispersed in the electrolyte solution or in water for unbiased solar water or overall water splitting can be collected by application of an external magnetic field for further use [41]. Fig. 6(a–c) shows magnetization *versus* magnetic field curves for pristine and surface-functionalized MoS_2_ nanoflakes. Pristine MoS_2_ is diamagnetic in nature (Fig. 6(a)); however, NiO NP–functionalized MoS_2_ and MoS_2_/NiO/MWCNT nanocomposite samples demonstrate ferromagnetic (FM) ordering at room temperature with 0.27 and 8.23 emu/g, respectively, of magnetism at the highest field of 7 T (Fig. 6(a) and (b)). Even at the highest field of 7 T, the magnetization does not saturate the MoS_2_/NiO/MWCNT nanocomposites. The variable temperature magnetic susceptibility (χ) measurements under 100 Oe FC and ZFC are presented in Fig. 5(d–f). The magnetic susceptibility (χ) measurements under FC and ZFC (Fig. 6(d)) for pristine MoS_2_ also show its diamagnetic nature. The negative Néel temperature obtained from the fitting is suggestive of antiferromagnetic (AFM) behavior contrasting the observed magnetization behavior. The susceptibility *versus* temperature plots for MoS_2_/NiO NPs and MoS_2_/NiO/MWCNT nanocomposite (Fig. 6(e) and (f)**)** start diverging at around room temperature, and demonstrates strong divergence below ~200 K, as opposed to the decrease/saturation in ferromagnets. These are the characteristic behavior of frustrated ferromagnetism. A frustrated system might arise when two types of magnetic behavior compete to coexist in the lattice*.* [42] In the susceptibility *versus* temperature plots, the ZFC measurements generally have a peak at blocking temperature. The ZFC curves of MoS_2_/NiO and MoS_2_/NiO samples show decrease in magnetic susceptibility and hence magnetic moment with decrease in temperature. In contrast to antiferromagnets, decrease in susceptibility in the present case is non-linear with decrease in temperature [43]. This trend may be due to the superposition of a linear term from the dominant AFM ordering and a non-linear term resulting from a blocking process. The subtraction of linear terms from corresponding ZFC curves results blocking temperature T_B_ = ~160 K and 175 K for MoS2/NiO and MoS2/NiO samples, respectively.

### 3.5 Hydrogen evolution reaction and electrochemical impedance spectroscopy (EIS) of different electrocatalysts in acidic and alkaline electrolytes

**Fig. 7:**
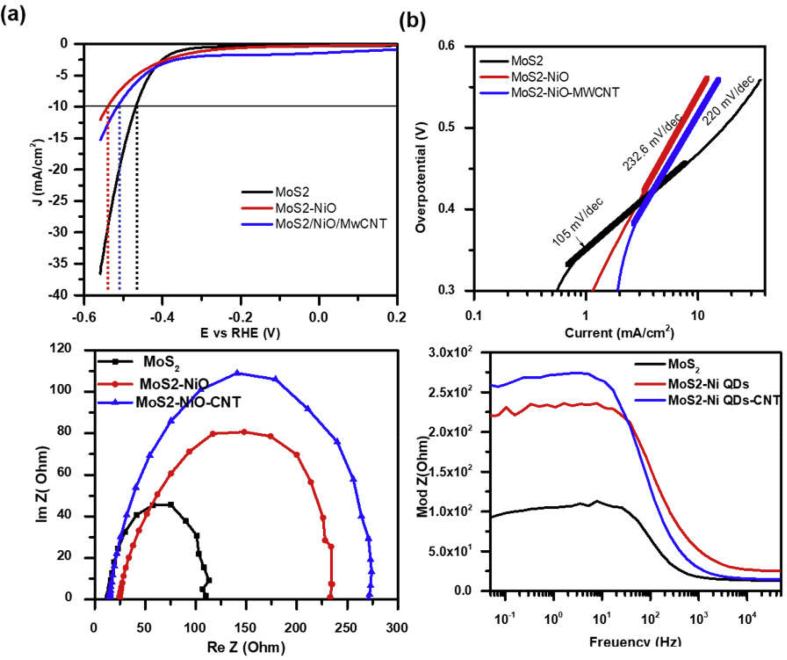
(a) Linear sweep voltammetry (LSV) and corresponding (b) Tafel plots for different MoS_2_ samples in 1 M NaOH solution. Electrochemical impedance spectroscopic (EIS) plots in the form of (c) Nyquist and (d) Bode plots.

The HER performance of different electrocatalysts are investigated in 1 M NaOH (pH: 12.5) and 0.5 M H_2_SO_4_ (pH: 0.3) electrolyte solutions using three electrode system, where electrodes made with the deposition of catalyst inks on the glassy carbon electrode was used as working electrode. The working electrode potential was varied from −0.6 to −1.6 V with respect to a SCE in alkaline medium and from +0.2 to −0.8 V in acidic medium. Working electrodes were prestabilized for 20–60 CV cycles with the scan rate of 10 mV/s before measurement of polarization curves. Fig. 7 shows HER and EIS measurements for different electrocatalyst samples in the alkaline medium. The polarization curve for MoS_2_ (black curve) has an onset potential (potential with current density > 1 mA/cm^2^) of ~0.35 V and shows a steep increase in the current density with an increase in the overpotential. The values of overpotential (η_10_), potential at which current density increases over 10 mA/cm^2^, and current density (I_0.5_), current density at 0.5 V potential, for bare MoS_2_ sample is ~0.45 V and 17 mA/cm^2^, respectively. The functionalization of MoS_2_ nanoflakes with NiO NPs decreases onset potential to 0.09 V and increases the value of η_10_ and I_0.5_ to ~0.53 V and ~7 mA/cm^2^ (red curve), respectively. Addition of MWCNTs into MoS_2_/NiO NPs system significantly changes its onset potential from negative (−0.9 V) to positive value (+0.15 V) with slight increase in η_10_ and I_0.5_ values (blue curve).

The Tafel slope is a useful parameter to evaluate the HER performance of electrocatalyst and provides information about the mechanism responsible for HER process [37,[44], [45], [46], [47], [48]]. The linear portion of Tafel plots [overpotential versus log (current density)] were fitted to the Tafel equation (η = blogj+a; where η is overpotential, j is current density, and b is the Tafel slope) that resulted in the Tafel slope. An electrocatalytic material with a smaller value of Tafel slope is considered as a better electrocatalyst because it increases hydrogen generation with a higher rate with an increase in the potential. The pristine MoS_2_ nanoflakes has a Tafel slope of ~105 mVdec^−1^ that increases to the values of 232.6 and 220 mVdec^−1^ for MoS_2_/NiO and MoS_2_/NiO/MWCNTs nanocomposite samples, respectively (Fig. 7(b)).

The working electrode kinetics at the electrode–electrolyte interface during HER operation was investigated using electrochemical impedance spectroscopy. Fig. 7(c) shows Nyquist (Re(Z) *versus* Im (Z)) plots for different electrocatalyst in the alkaline medium. The Nyquist plot demonstrates one-to-one correspondence with HER activity. For example, a better HER active material corresponds to a semicircle with a smaller radius in the Nyquist plot. This indicates a smaller charge transfer resistance (R_ct_) and fast shuttling of electrons during HER process. Here, pristine MoS_2_ forms the least semicircle, whereas MoS_2_/NiO/MWCNT nanocomposite corresponds to the largest. The Bode plot that presents the modulus of impedance as a function of log of frequency also supports HER measurements and Nyquist plots. Here, the modulus of impedance is the maximum for MoS_2_/NiO/MWCNT nanocomposite sample for every frequency in the range of 10 mHz–100 Hz. The impedance of MoS_2_, MoS_2_/NiO, and MoS_2_/NiO/MWCNT samples are 14, 16, and 25 Ωs, respectively, at higher frequency (10^4^ Hz) (Fig. S3(a)). These impedance values are used for iR correction of corresponding polarization curves shown in Fig. 7(a)**.**

**Fig. 8:**
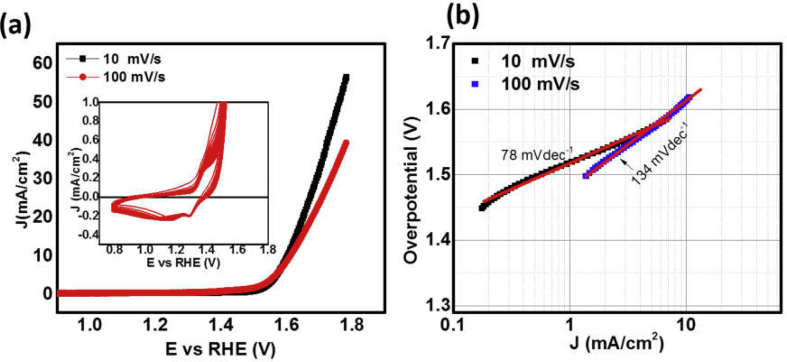
(a) Linear sweep voltammetry (LSV) curves and (b) corresponding Tafel plots for anodic current demonstrating oxygen evolution reaction (OER) from working electrode made of MoS_2_/NiO NPs in alkaline medium with the scan rates of 10 and 100 mV/s. (Inset) Twenty cycles of CV curves in non-Faradaic region (0.8–1.2 V *vs* RHE) demonstrating onset potential of 1.5 V *vs* RHE.

The working electrode made with the MoS_2_/NiO NPs electrocatalyst was also tested for OER performance in the alkaline electrolyte. Potential at the working electrode was scanned in the range of −0.2 to +0.8 V (vs SCE) with the scan rates of 10 and 100 mV/s (Fig. S4). Polarization curves were recorded after 20 cycles of prestabilization and translated to RHE potential (Fig. 8(a)). Here, onset potential is 1.5 V and peak current density ~58 mA/cm^2^ at an overpotential of 1.8 V. Higher scan rate exhibits comparatively lower values onset potential and current density. Tafel plots for the polarization curves measured with 10 and 100 mV/s scan rates have 78 and 134 mVdec^−1^, respectively, of Tafel slopes for OER (Fig. 8(b)). These measurements show that scan rate significantly affects catalytic activity at electrode–electrolyte interface. Effects of scan rate is opposite for electrode biased negatively or positively with respect to reference electrode. Inset of Fig. 8(a) and Fig. S5 demonstrate CV cycles for adsorption of OH^−^ ions and release of O_2_ molecules from electrolyte surface.

**Fig. 9:**
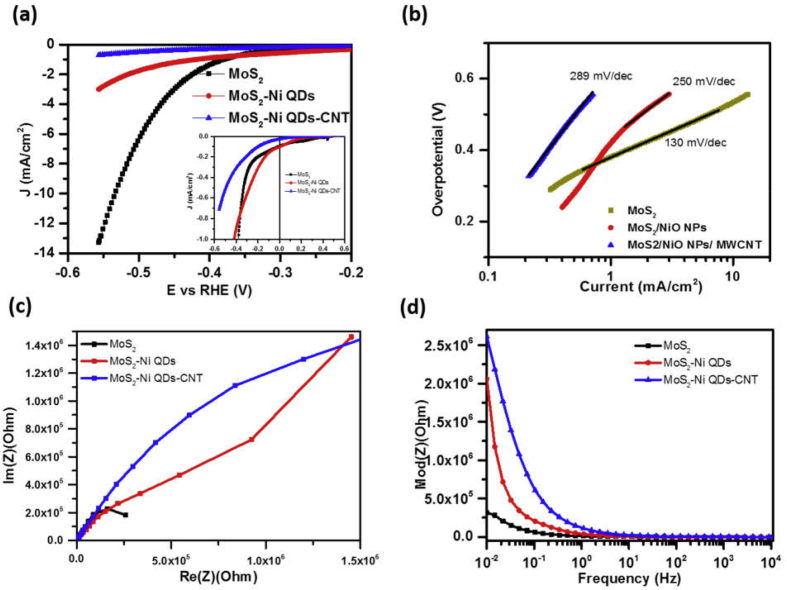
(a) Linear sweep voltammetry (LSV) and corresponding (b) Tafel plots for different MoS_2_ samples in 0.5 M H_2_SO_4_ solution. Electrochemical impedance spectroscopic (EIS) plots in the form of (c) Nyquist and (d) Bode plots. EIS measurements were done at VRHE = 0.22 V.

Fig. 9 presents the HER and the EIS measurements of pristine and surface-functionalized MoS_2_ in 0.5 M H_2_SO_4_ electrolyte. Here, in the acidic medium, pristine MoS_2_ demonstrates best HER performance with onset potential of 0.4 V (Fig. S8(b)) @ 1 mA/cm^2^ of current density. It results for maximum current density of 13.5 mA/cm^2^ at 550 mV overpotential. Functionalization of MoS_2_ with NiO NPs slightly increases onset potential, but significantly decreases cathodic current from 13.5 mA/cm^2^ to 3 mA/cm^2^. Addition of MWCNT into NiO NP–functionalized MoS_2_ system further decreases cathodic current from 3 mA/cm^2^ to 0.77 mA/cm^2^ at an overpotential of 550 mV. The corresponding Tafel plots (Fig. 9(b)**)** show that the value of Tafel slope increases with the surface functionalization of MoS_2_ nanoflakes and have values of 130, 250, and 289 mV/dec for pristine MoS_2_, MoS_2_/NiO NPs, and MoS_2_/NiO NPs/MWCNT nanocomposite electrocatalysts, respectively. The Nyquist and Bode plots (Fig. 9(c) and (d)) also show one-to-one correspondence with polarization curves and support HER results. The stability tests also show that pristine MoS_2_ sample exhibits higher cathodic current density and better stability in the acidic electrolyte (Fig. S6 and S7). Performance parameters for different electrocatalysts in alkaline and acidic media are presented in Table 1, Table 2, respectively.

**Table 1:** Performance parameters of different electrocatalysts in alkaline medium.

Parameters	MoS_2_	MoS_2_/NiO	MoS_2_/NiO/MWCNT
Onset potential (V)	0.35	0.09	0.15
η_10_ (V)	0.45	0.53	0.51
I_0.5_ (mA/cm^2^)	18	7	9
Tafel slope (mV/dec)	105	232.6	220
Re (Z) at Im (Z) = 0 (Ohm)	109	234	272
Mod Z at 0.1 Hz (Ohm)	100	227	265
Mod Z at 10^4^ Hz (Ohm)	13	26	15

**Table 2:** Performance parameters of different electrocatalysts in acidic medium.

Parameters	MoS_2_	MoS_2_/NiO	MoS_2_/NiO/MWCNT
Onset potential (V)	0.37	0.42	–
η_10_ (V)	0.54	–	–
I_0.5_ (mA/cm^2^)	6.52	1.84	0.53
Tafel slope (mV/dec)	130	250	289
Re (Z) at Im (Z) = 0 (Ohm)	–	–	–
Mod Z at 0.1 Hz (kOhm)	53	212	613
Mod Z at 10^4^ Hz (Ohm)	11	63	97

**Fig. 10:**
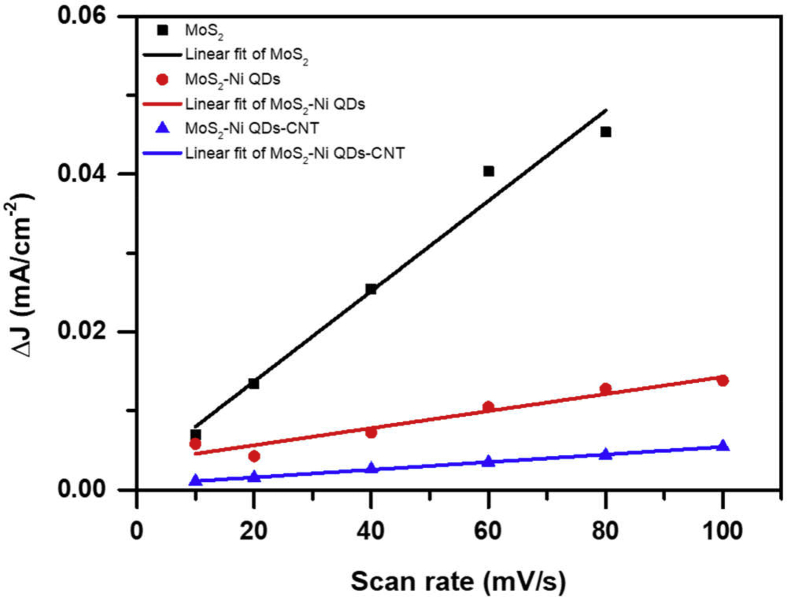
Change in current density *versus* scan rate graph for the estimation of ESCA of different electrocatalysts in acidic medium.

Electrochemically active surface area (ECSA) is another important parameter for evaluating the HER performance of an electrocatalyst. A larger active surface area provides larger hydrogen adsorption sites and hence larger hydrogen generation. The ESCA values for different electrocatalysts in acidic medium were estimated by the measurement of double-layer capacitance in a non-Faradaic region. CV curves (Fig. S9), recorded at different scan rates for each sample in the acidic medium, were used for the calculation of electrochemical double-layer capacitance (C_dl_). The current density difference at the intermediate overpotential in the non-Faradaic range was plotted against the scan rates (Fig. 10). Half of the slope of the linear fit for difference in current density (ΔJ) versus scan rate gives the value of C_dl_. The ECSA values are estimated by the ratio of double-layer capacitance to the specific capacitance (Cs) of an atomically smooth MoS_2_ nanosheet (Cs = ~60 μF/cm^2^) [37]. The calculated ECSA values 72, 14, and 6 for MoS_2_, MoS_2_/NiO NPs, and MoS_2_/NiO/MWCNT nanocomposite samples, respectively, are in accordance with the observed HER result in the acidic electrolyte.

The HER is a two-electron exchange reaction mechanism, where electrocatalysts are required to overcome the energy barriers in each step. The generally accepted pathways for HER in electrolyte solution are adsorption/desorption of a hydrogen intermediate ($H_{ad}$) through either Volmer–Heyrovsky or the Volmer–Tafel mechanism [[49], [50], [51]]. In the first step (Volmer step), hydrogen ions get adsorbed on the active sites of catalysts following both mechanisms. In the second step, either two hydrogen atoms adsorbed on the active sites get recombined (Tafel step) to generate a hydrogen molecule or a hydrated proton directly combines with an adsorb hydrogen atom through transfer of an electron from the electrode material (Heyrovsky step). The mechanism and pathways of hydrogen generation in alkaline and acidic media are presented as follows:

In alkaline medium,

(1) $$2H_{2}O+2e\to H_{2\left(g\right)}+2OH_{aq}^{-}$$

Reaction presented in equation 1 passes through the following three steps:

$$2H_{2}O+2e^{-}\to 2H_{ad}+2OH^{-}\text{(Volmer}\;\;\text{step}\;\text{of}\;\text{water}\;\text{dissociation)}$$

$$H_{2}O+H_{ad}+e^{-}\to H_{2\left(g\right)}+OH^{-}\;\text{(Heyrovsky step)}$$

$$2H_{ad}\to H_{2\left(g\right)}\text{(Tafel step)}$$

In acidic medium,

(2) $$2H_{aq}^{+}+2e^{-}\to H_{2\left(g\right)}$$

Generation of hydrogen in acidic medium following equation 2 passes through the following three steps:

$$H^{+}+e^{-}\to H_{ad}\;\text{(Volmer step)}$$

$$H_{ad}+H^{+}+e^{-}\to H_{2}\text{(Heyrovsky step)}$$

$$2H_{ad}\to H_{2\left(g\right)}\text{(Tafel step)}$$

**Fig. 11:**
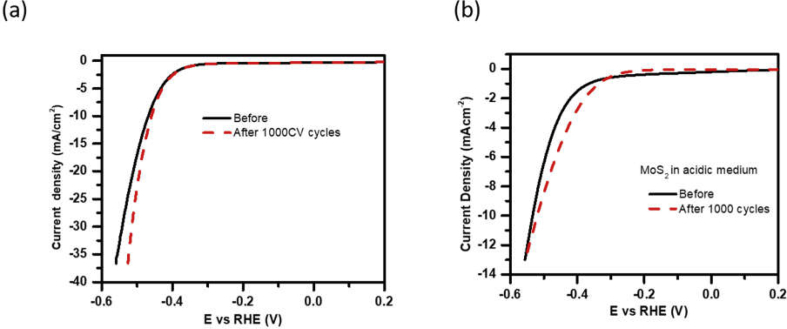
Polarization curves of MoS_2_ in (a) alkaline and (b) acidic electrolyte media before (black curve) and after (red curve) 1000 CV cycles.

For the stability test, MoS_2_ working electrode was scanned for 1000 CV cycles in the alkaline and acidic media in the potential range of 0.2 to −0.6 V (RHE) with the scan rate of 100 mV/s. The polarization curves were measured after 1000 CV cycles (red dashed curve) and presented in Fig. 11 along with corresponding initial polarization curves. These measurements show that MoS_2_ electrocatalyst is stable in both media, but it has better stability and better performance in the alkaline medium over the acidic medium. Table 3 presents some of the previous reports on electrocatalytic hydrogen generation from MoS_2_ in acidic and alkaline media.

**Table 3:** Comparison of the catalytic performances involving MoS_2_-based materials for HER.

Catalyst	Electrolyte Medium	Onset potential (mV)	Overpotential (mV)	Tafel slope (mV/dec)	Current density (mA/cm^2^)	Reference
MoS_2_ thin film	0.5 M	–	–	140	0.6 × 10^−2^	[52]
	H_2_SO_4_					
MoS_2_/graphene	0.5 M	–	30 mV	67.4	0.14	[53]
Nanosheets	H_2_SO_4_	–				
MoS_2_	0.5 M	–	170	60	–	[54]
Nanosheets/strained	H_2_SO_4_					
S vacancies						
Oxygen	0.5 M	120	300	55	126.5	[55]
Incorporated MoS_2_	H_2_SO_4_					
Nanosheets						
MoS_2_ nanosheets	0.5 M	–	–	68	3.89 × 10^−2^	[56]
	H_2_SO_4_					
MoS_2_ monolayer	0.5 M	100	–	73	2.45 × 10^−2^	[57]
Flakes	H_2_SO_4_					
Hierarchical MoS_2_	0.5 M	50	167	70	3.6 × 10^−2^	[58]
Nanosheets	H_2_SO_4_					
MoS_2_/Ni_3_S_2_ heterostructures	1 M KOH	50	110	83	–	[59]
Ni-doped MoS_2_ nanosheets	1 M KOH	–	98	60	0.98	[60]
Ni(OH)_2_/MoS_2_ heterostructures	1 M KOH	20	80	69	–	[61]
Few-layered MoS_2_ nanosheets	0.5 M	370	540	130	13	This work
	H_2_SO_4_					
Few-layered MoS_2_ nanosheets	1 M NaOH	350	450	105	37	This work

## 4 Conclusion

In summary, we presented a scalable synthesis method for production of few-layered MoS_2_ nanosheets and their surface functionalization using NiO NPs and MWCNTs and reported HER activity of pristine and surface functionalized few-layered MoS_2_ nanoflakes in acidic and alkaline electrolytes. Surface functionalization converts diamagnetic MoS_2_ nanoflakes into FM electrocatalysts. For bare MoS_2_, the values of overpotential (η_10_) in alkaline and acidic media are 0.45 and 0.54 V, respectively. Similarly, the values of current density at 0.5 V overpotential are 27 and 6.2 mA/cm^2^ in alkaline and acidic media, respectively. The surface functionalization acts adversely in the both alkaline and acidic media. Performance parameters such as onset potential, η_10,_ current density, Tafel slope of bare, and surface-functionalized MoS_2_ nanosheets in alkaline media are higher as compared with acidic electrolyte. Similarly, for every electrocatalysts, impedances at higher and lower AC frequencies are lower for alkaline medium over that of acidic medium. MoS_2_ nanosheets functionalized with NiO NPs also demonstrated excellent performance for OER with anodic current of ~60 mA/cm^2^ and Tafel slope of 78 mVdec^−1^ in alkaline medium.

## Conflicts of interest statement

The authors declare no competing financial interest.
