# Supplementary material for: Hydrogen evolution reaction from bare and surface-functionalized few-layered MoS2 nanosheets in acidic and alkaline electrolytes
Source: Mater Today Chem. 2019 Dec;14:100207. doi: 10.1016/j.mtchem.2019.100207 (PMC6936932; doi:10.1016/j.mtchem.2019.100207)
Supplement: Multimedia component 1 [file mmc1.docx]

**Supporting Information**

**Hydrogen evolution reaction from bare and surface functionalized few-layer MoS_2_ nanosheets in acidic and alkaline electrolytes**

^1,3^Bo Lai^#^, ^1,3^S.C. Singh^#^,^2^Jasleen K. Bindra^#^ ,^3^C.S. Saraj, ^3^Abhishek Shukla, ^4^T.P. Yadav,^3^Weili Wu, ^5^Stephen A. McGill,^2^N.S. Dalal,^2,6^Amit Srivastava*and ^1,3^Chunlei Guo*

^1^*The Institute of Optics, University of Rochester, Rochester, NY 14627, USA*

*^2^Department of Chemistry and Biochemistry, Florida State University, Tallahassee, Florida 32306, USA*

*^3^Changchun Institute of Optics, Fine Mechanics and Physics, Chinese Academy of Sciences, Changchun 130033, China*

*^4^Department of Physics, Institute of Science, Banaras Hindu University, Varanasi-222005, India*

*^5^National High Magnetic Field Laboratory, Florida State University, Tallahassee, FL, 30201, USA*

*^6^Department of Physics, TDPG College, VBS Purvanchal University, Jaunpur-222001,India*

*Corresponding authors: [amitrac@gmail.com](mailto:amitrac@gmail.com); [guo@optics.rochester.edu](mailto:guo@optics.rochester.edu);

^#^First author and authors with equal contribution

Note:

The authors declare no competing financial interest

**
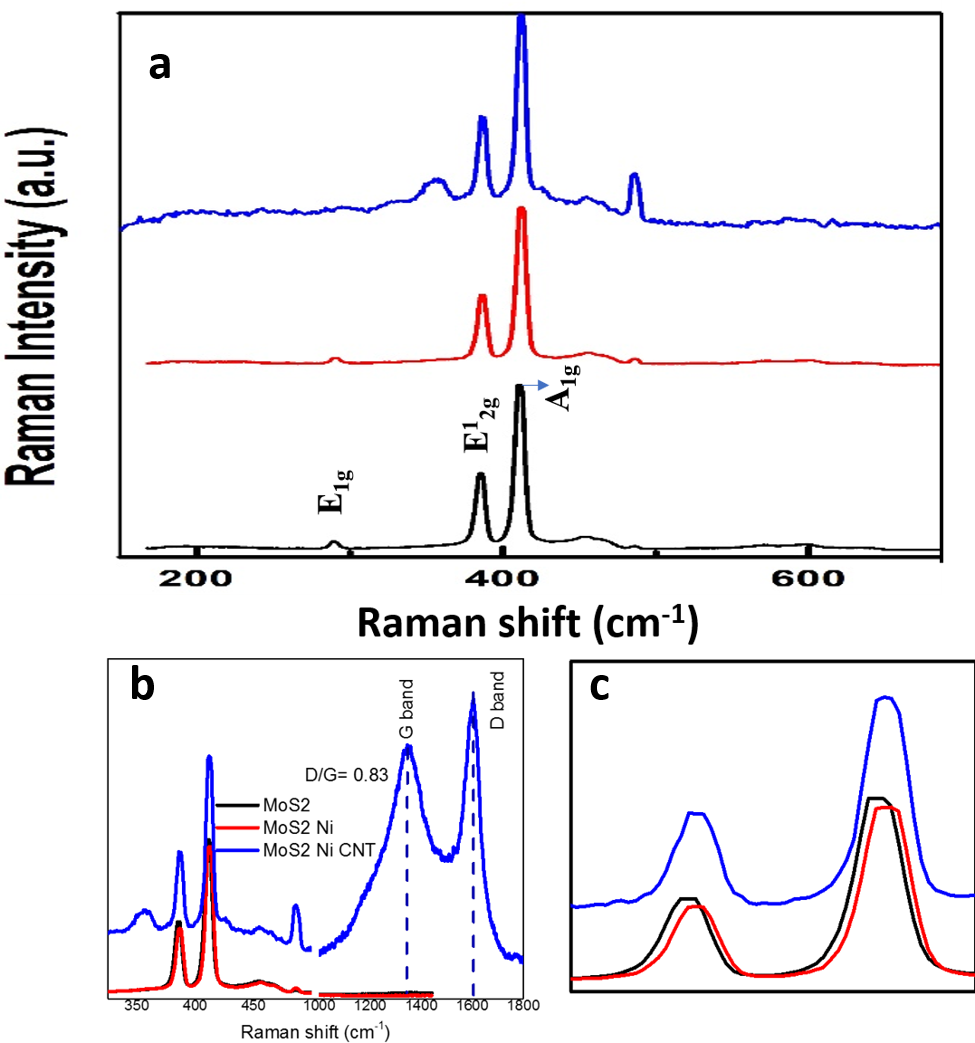
**

**Fig. S1: (a)** Raman spectra of pristine MoS_2_ (black curve), NiO NPs functionalized MoS_2_ (red curve), and MoS_2_/NiO/MWCNT composite (blue curve). (b) Comparative Raman spectra of three electrolytes alongwith D and G band of MWCNT, (c) Enlarged view of (b) in the range of 350 to 420 cm^-1^

**
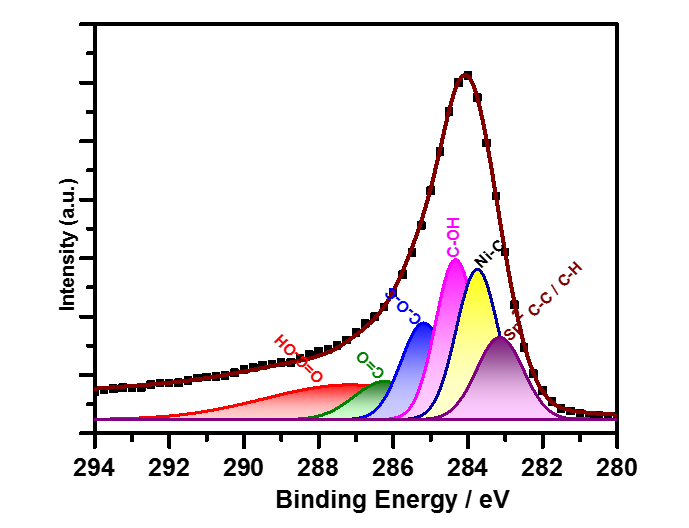
**

**Fig. S2:** XPS spectrum of MoS_2_/ NiO NPs/ MWCNTs in the energy range of 280-294 eV for carbon. XPs peak is deconvoluted into different species present on the surface of CNT.

**
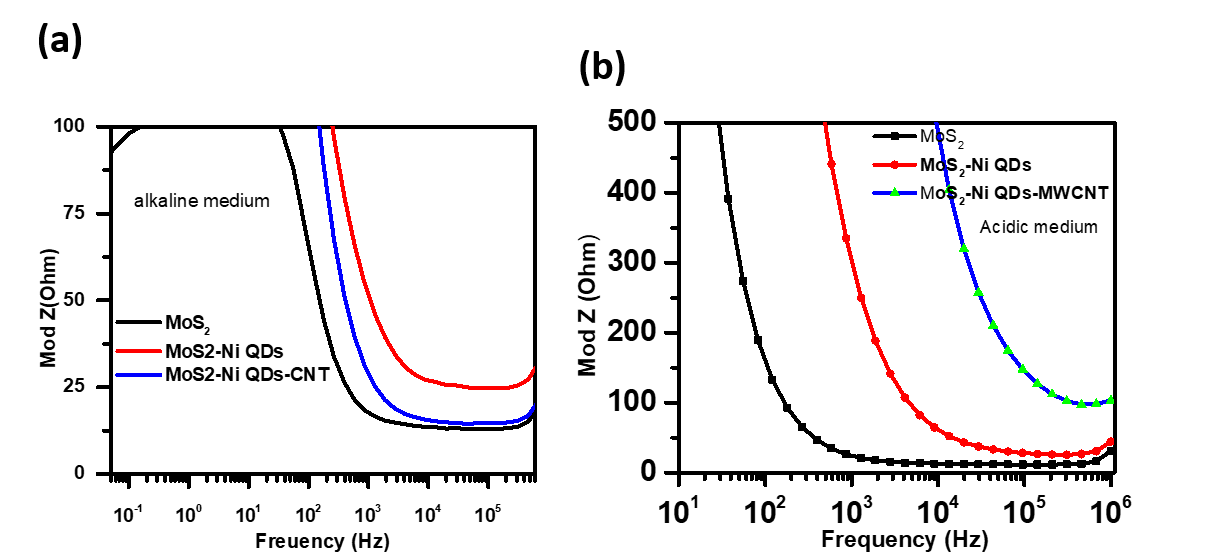
**

**Fig. S3:** Enlarged view of Bode plots for different electrolytes in the frequency range of 10Hz to 10 MHz in (a) basic and (b) acidic electrolytes

**
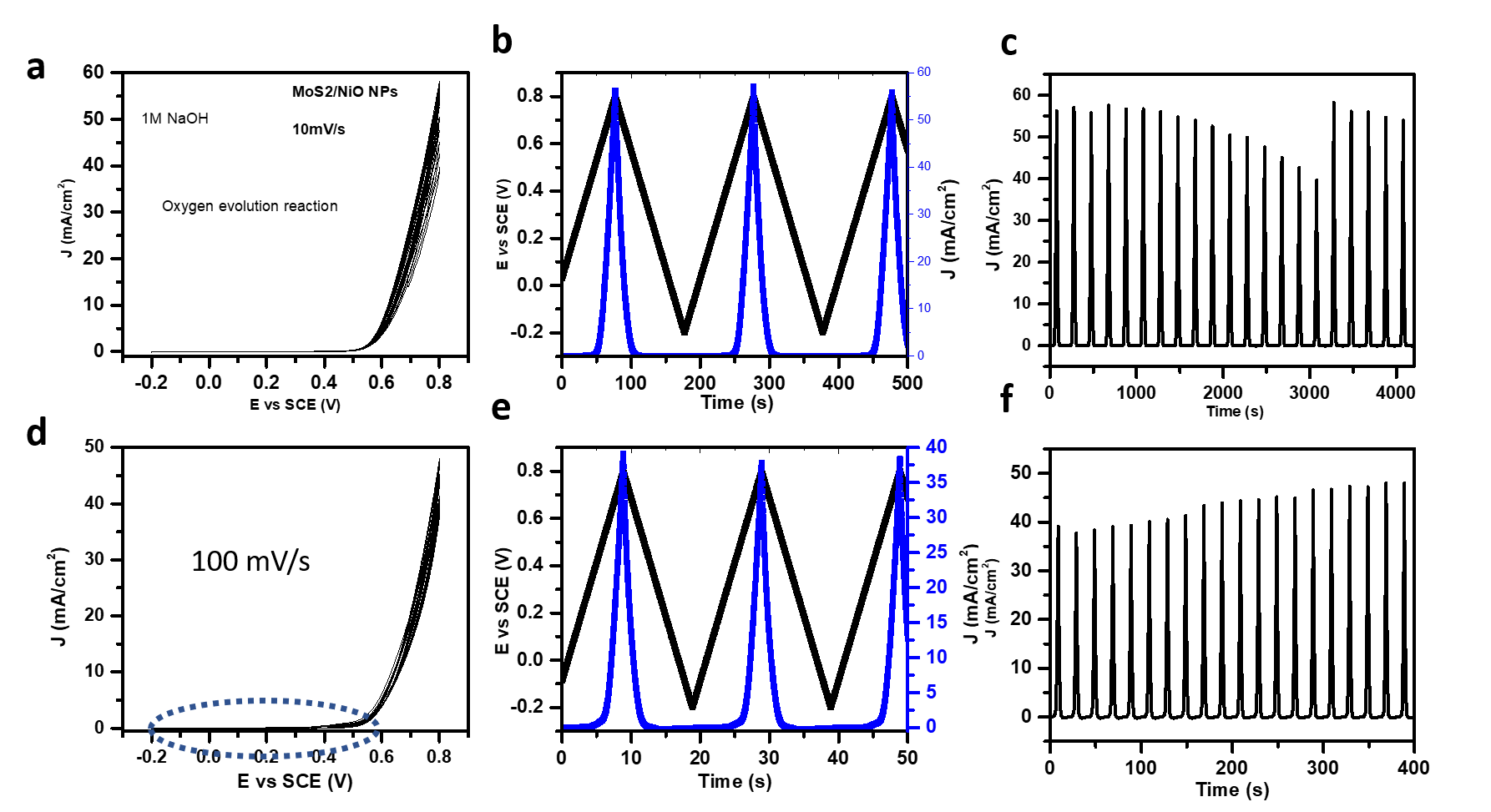
**

**Fig. S4:** Twenty cycles of oxygen evolution reaction (positive biasing of working electrode with respect to SCE) of MoS_2_/NiO NPs with scan rates of (a) 10 mV/s and (d) 100 mV/ s in the potential range of -0.2 to 0.8 (vs SCE). Corresponding timing diagram for potential applied (vs SCE) and anodic current density for 2.5 cycles. Variation in the anodic current with time for twenty cycles when potential at working electrode is varied in the range of -0.2 to 0.8 V (vs SCE) with scan rates of (c) 10 mV/s and (f) 100 mV/s.

**
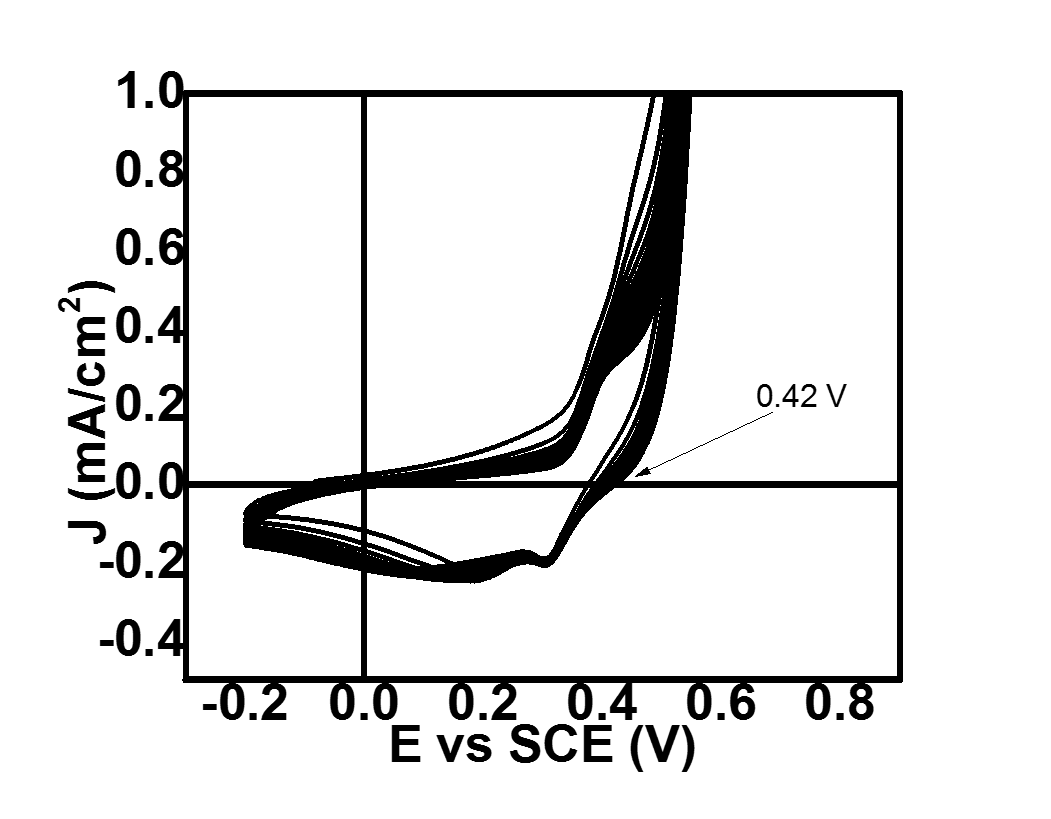
**

**Fig. S5:** Twenty cycles of CV curve in the potential range of -0.2 to 0.6 V (blue encircled in Fig. S3 (d)) (non-Faradaic region) to demonstrate oxidation, reduction reaction at electrode.

**
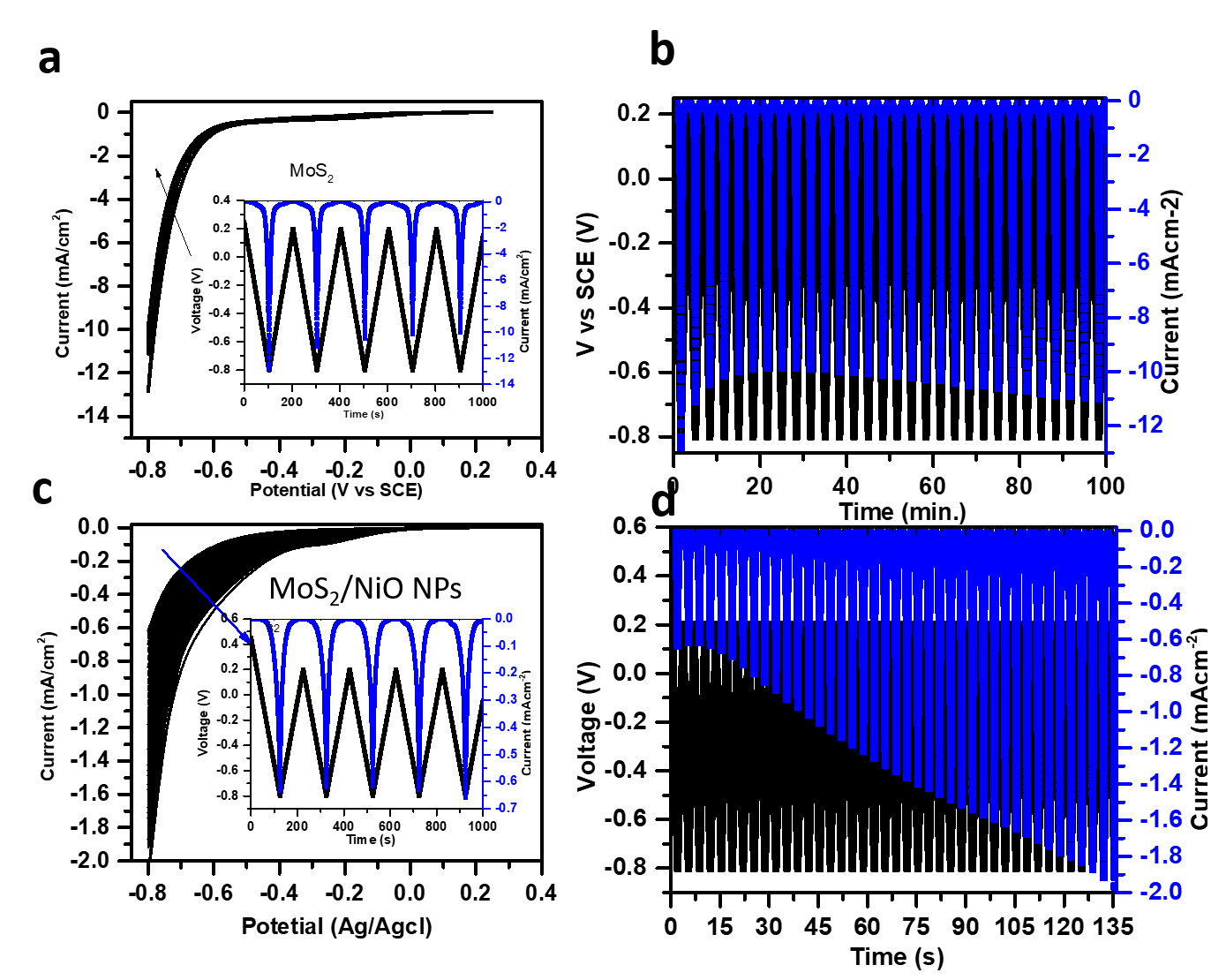
**

**Fig. S6:** Pre-stabilization test for (a,b) MoS2 and (c,d) MoS2/NiO NPs in acidic electrolyte with the scan rate of 10 mV/s .

**
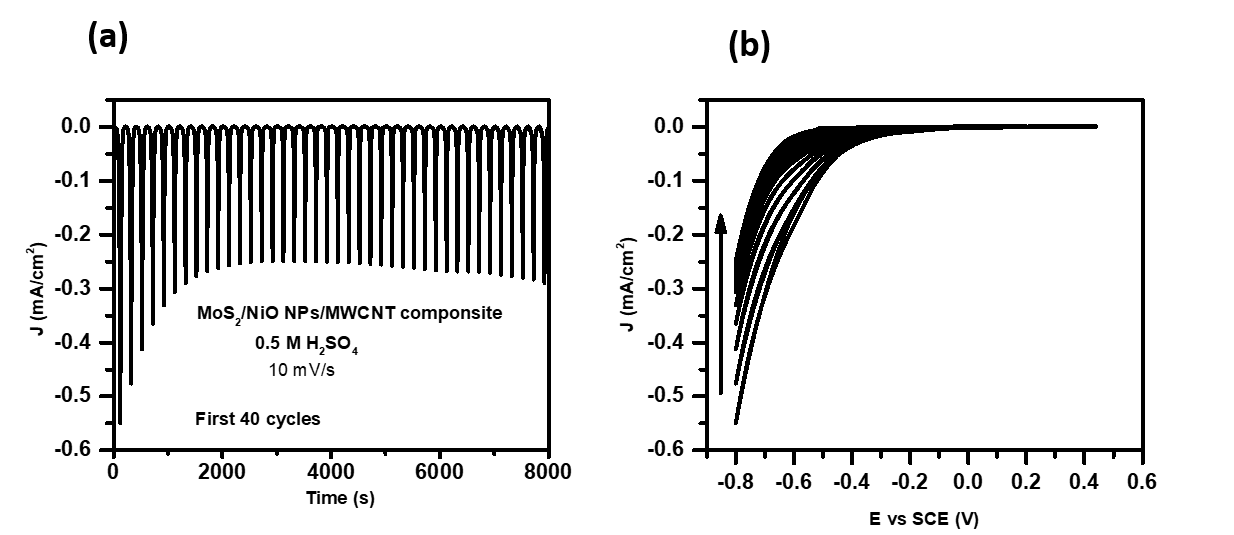
**

**Fig. S7:** Pre-stabilization test for MoS2/NiO NPs/MWCNT in acidic electrolyte with the scan rate of 10 mV/s.

**
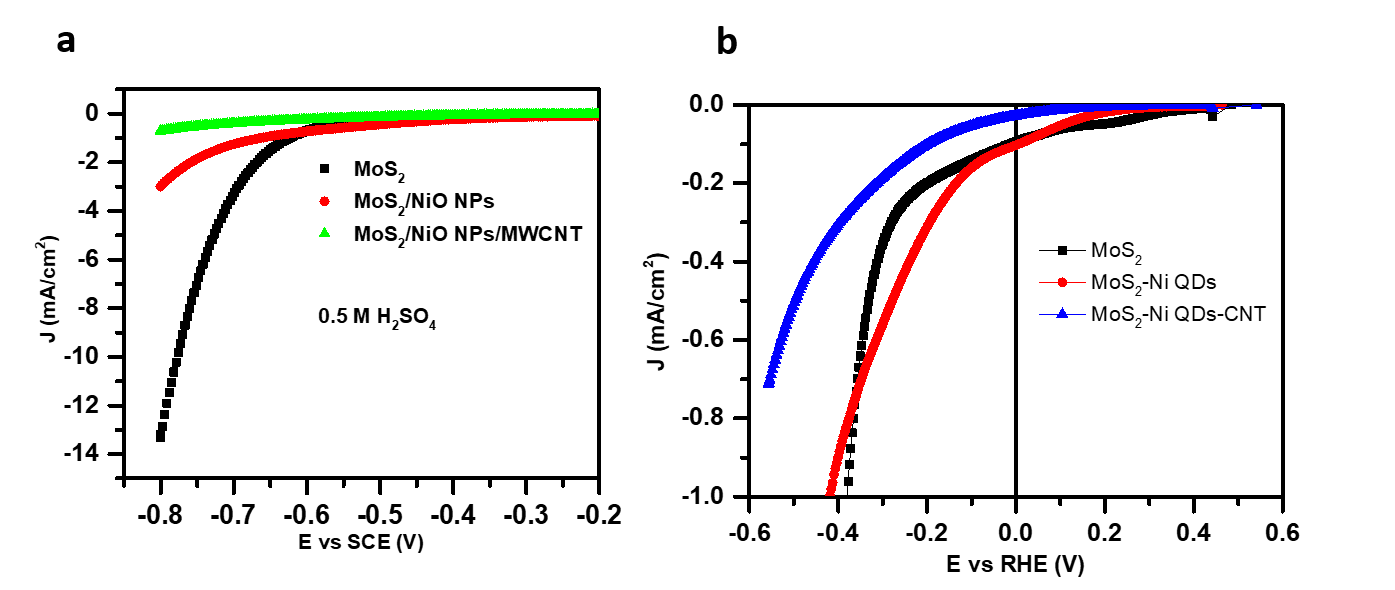
**

**Fig. S8: (a)** LSV curves after 40 cycles of pre-stabilization with potential in the range of -0.2 to -0.8 V (vs SCE). (b) Enlarge view of LSV curves for different electrolytes near zero potential (*vs* RHE) in acidic electrolyte

**
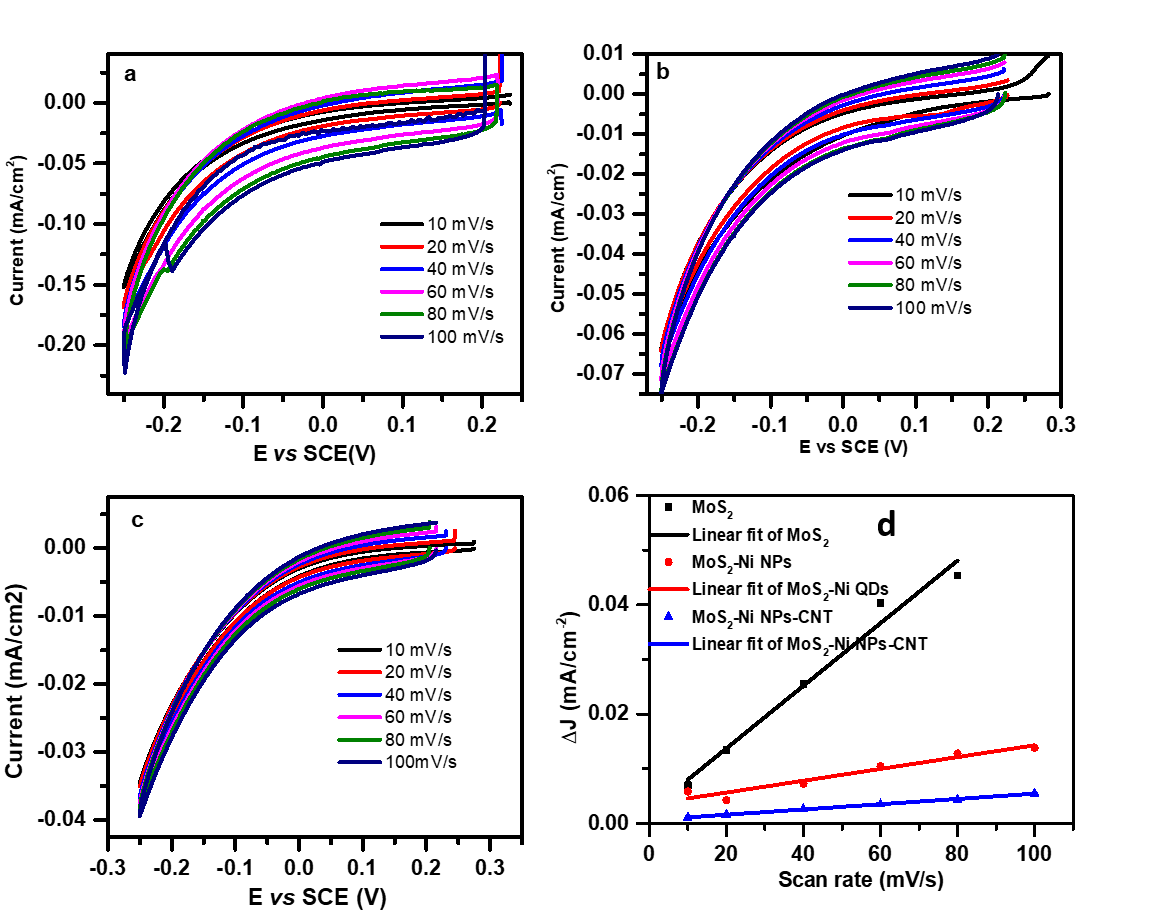
**

**Fig. S9:** CV curves for (a) MoS_2_, (b) MoS_2_/NiO, and (c) MoS_2_/NiO/MWCNT electrocatalysts in the potential range of +0.2 to -0.25 V (SCE) (Non-Faradaic region) in acidic medium with different scan rates. (d) Variation of change in current density at intermediate potential with scan rate for the estimation of ESCA.
